# 

**Published:** 1872-11

**Authors:** 


A NUMBER of books, etc., have been received, and will be noticed in the next number of the JOURNAL.

BY A MISTAKE in the press room there was a failure to strike a sufficient quantity of the November number to supply our subscribers, and consequently there has been a change in the arrangement announced in the *Daily Herald* a few days since, which contemplated the issue of the November and December numbers under the same cover. Our patrons may be assured that they shall sustain no loss, but that some satisfactory adjustment will be made.

THE PUBLISHERS.

